# Higher risk of 2-year cup revision of ceramic-on-ceramic versus ceramic-on-polyethylene bearing: analysis of 33,454 primary press-fit total hip arthroplasties registered in the Dutch Arthroplasty Register (LROI)

**DOI:** 10.1177/11207000211064975

**Published:** 2022-01-02

**Authors:** Justin van Loon, Inger N Sierevelt, Anneke Spekenbrink-Spooren, Kim TM Opdam, Rudolf W Poolman, Gino MMJ Kerkhoffs, Daniël Haverkamp

**Affiliations:** 1 Xpert Clinics Orthopedie Amsterdam, The Netherlands; 2 Department of Orthopaedic Surgery, Amsterdam Movement Sciences, Amsterdam UMC, Academic Medical Centre, University of Amsterdam, Amsterdam, The Netherlands; 3 Department of Orthopaedic Surgery, Tergooi, Hilversum, The Netherlands; 4 Department of Orthopaedic Surgery, Spaarne Gasthuis Academy, TM Hoofddorp, The Netherlands; 5 Landelijke Registratie Orthopedische Implantaten (LROI), ‘s-Hertogenbosch, The Netherlands; 6 Department of Orthopaedic Surgery, Leiden University Medical Centre, Leiden, The Netherlands; 7 Department of Orthopaedic Surgery, OLVG, Amsterdam, The Netherlands

**Keywords:** Aseptic loosening, bearing, early revision, press-fit, primary stability, total hip arthroplasty

## Abstract

**Background and purpose:**

The influence of bearing on short-term revision in press-fit total hip arthroplasty (THA) remains under-reported. The aim of this study was to describe 2-year cup revision rates of ceramic-on-ceramic (CoC) and ceramic-on-polyethylene (CoPE).

**Patients and methods:**

Primary press-fit THAs with one of the three most used cups available with both CoC or CoPE bearing recorded in the Dutch Arthroplasty Register (LROI) were included (2007–2019). Primary outcome was 2-year cup revision for all reasons. Secondary outcomes were: reasons for revision, incidence of different revision procedures and use of both bearings over time.

**Results:**

2-year Kaplan-Meier cup revision rate in 33,454 THAs (12,535 CoC; 20,919 CoPE) showed a higher rate in CoC (0.67% [95% CI, 0.54–0.81]) compared to CoPE (0.44% [95% CI, 0.34–0.54]) (*p* = 0.004). Correction for confounders (age, gender, cup type, head size) resulted in a hazard ratio (HR) of 0.64 [95%CI, 0.48–0.87] (*p* = 0.019). Reasons for cup revision differed only by more cup revision due to loosening in CoC (26.2% vs.1 3.2%) (*p* = 0.030). For aseptic loosening a revision rate of 0.153% [95% CI, 0.075–0.231] was seen in CoC and 0.058% [95%CI 0.019–0.097] in CoPE (*p* = 0.007). Correction for head size resulted in a HR of 0.475 [95% CI, 0.197–1.141] (*p* = 0.096). Incidence of different revision procedures did not differ between bearings. Over time the use of CoPE has increased and CoC decreased.

**Conclusions:**

A higher 2-year cup revision rate in press-fit THA was observed in CoC compared to CoPE. Cup loosening was the only significantly different reason for revision and seen more often in CoC and mostly aseptic. Future randomised controlled trials need to confirm causality, since the early cup revision data provided has the potential to be useful when choosing the bearing in press-fit THA, when combined with other factors like bone quality and patient and implant characteristics.

## Introduction

The literature suggests that the main reason for late revisions in press-fit total hip arthroplasty (THA) is aseptic loosening of the cup caused by wear-induced osteolysis of polyethylene (PE) liners.^1–7^ Despite the process of cross-linking to improve wear rates, ceramic-on-ceramic (CoC) still remains one of the best options to overcome liner wear. CoC shows wear rates below 0.001 mm/year compared to 0.072 mm/year in conventional ceramic-on-polyethylene (CoPE), 0.042 in metal-on-highly cross-linked PE (MoHXLPE) and 0.030 mm/year in ceramic-on-highly cross-linked PE (CoHXLPE).^8^ Despite this, combinations of polyethylene liners with a ceramic head remain the most used bearing in THA in The Netherlands.^9^ The influence of bearing on infection, dislocation and aseptic loosening in explanations of early revision of the cup remains under-reported.^1,7,10^ No differences in periprosthetic joint infection between bearings were observed at 6 months; nevertheless, at 15 years significantly less infections were seen in CoC.^11^ Revision because of dislocation was seen less in CoC compared to CoPE at 9 years, due to a bigger head size in CoC.^12^ Focusing on aseptic loosening of the cup, higher early revision rates in CoC are seen, which might be caused by the bearing itself.^13^ In stiff CoC bearings, a less physiologic load transfer to the bone-implant interface is seen, resulting in increased micromotion.^14,15^ This jeopardises osseointegration and following transition to secondary stability due to failure of ingrowth and can cause aseptic loosening of the cup. Evidence of hard-on-hard bearings on this process is still limited.^16,17^ While life expectancy and prevalence of THA increase, there has been a shift to younger age groups of patient over the last decades.^18^ This emphasises the need for research to find an implant with low wear and complication rates and long survival.

Our primary goal was to describe the 2-year cup revision rates of CoC and CoPE. Following that, the reasons for revision, incidence of different revision procedures and use of both bearings over time will be described.

Our hypothesis was that a higher early revision rate may be observed in CoC compared to CoPE. We expect that reasons for revision will differ between both groups, with more aseptic cup loosening in CoC, and a decrease in use of CoC over time.

## Materials and methods

### Data sources

The Dutch Arthroplasty Register (LROI) is a nationwide population-based registry that has recorded information on joint arthroplasties in the Netherlands since 2007. It was initiated by the Netherlands Orthopedic Association (NOV) and had a completeness up to 99% for primary THAs and 98% for hip revision arthroplasties in 2020.^19^ The LROI database provides information on patient characteristics, surgical procedure and prosthesis characteristics, registered by all the hospitals in The Netherlands at the time of the primary operation by barcode scanning. The information about the prosthesis characteristics is supplied by implant manufacturers and distributors in The Netherlands, using a registration form. The vital status of all patients is obtained from Vektis, the national health insurance database in The Netherlands. An opt-out system is used by the LROI to obtain informed consent by patients.

### Data collection and patients

Eligible patients were registered in the LROI as having received a primary press-fit THA with either a CoC or CoPE bearing, from 2007 until the end of the follow-up period on 31 December 2019. Only the 3 most frequently implanted cup types available with both CoC and CoPE bearing were selected, since a selection of more cups would have resulted in more heterogeneity in cup type and thereby statistically may have interfered with our goal to analyse the effect of bearing type on outcomes. Moreover, most cup types registered in the LROI are not available with both CoC and CoPE bearing. The indications for THA in this study were primary osteoarthritis (OA), osteonecrosis, acute femoral neck fracture and secondary osteoarthritis due to hip dysplasia. All press-fit THAs included for this study were defined as a procedure in which the cup was a press-fit uncemented implant, with every conventional stem. Since polyethylene liners are mainly differentiated by their wear characteristics, which will not occur within a 2-year follow-up, all kinds of liners, either conventional, (highly) cross-linked or other PE based liners, were amalgamated into 1 group, named as CoPE throughout this paper. The patient demographics recorded were age, gender, American Society of Anesthesiologists (ASA) score, body mass index (BMI), indication for THA (categorised as primary OA or other) and prior operation to the hip. Prosthesis characteristics recorded were cup size, head size, stem size, and surgical approach. Charnley Classification and smoking were also recorded, but only recorded in the LROI since 2014. We chose a minimal observation period of 2 years as the cut off point for revision rate, as previous radiostereometric analysis (RSA) studies suggest that early cup migration, which can result in loosening, is mostly seen in the first 6 months after implantation and stabilises within 2–3 years.^20,21^

### Primary outcome

Primary outcome was the early cup revision rate for all reasons within the first 2 years after implantation. This outcome was analysed when comparing CoC with CoPE in the 3 most used cup types available with both CoC and CoPE bearing in the LROI. When indicated, this outcome was corrected for patient factors (age, ASA score, gender), indication for surgery, surgical approach, cup type, cup size or head size. Since a minimal available follow-up of 2 years was necessary for this outcome, only those THAs implanted from the beginning of the LROI in 2007 until 31 December 2017 were selected for this research question.

### Secondary outcomes

Secondary outcomes were reasons for early cup revision, incidence of revision procedures performed and use of both bearings over time from 2007 till 2019. Separately from the reasons for early cup revision, the 2-year revision rate for aseptic loosening of the cup was calculated. Aseptic cup revision was defined as a procedure where at least the cup was exchanged or removed, without signs of infection as stated in the LROI. When a revision of the cup was performed, this procedure was scored in the LROI as either an isolated cup revision, total revision or resection arthroplasty according to Girdlestone. The aforementioned secondary outcomes were compared between CoC and CoPE in the 3 most used cup types available with both CoC and CoPE bearing in the LROI.

### Statistical analysis

Revision of the cup for all reasons was the endpoint of the primary analysis. 2-year revision rates were calculated for both CoC and CoPE using Kaplan-Meier analysis, as mortality was not considered a competing risk at this short term.^22^ Comparison of the revision rates was performed by use of a Log Rank test. Crude as well as multivariable Cox proportional Hazard models were used to calculate Hazard Ratios (with 95% confidence interval [CI]) for early revision of CoPE compared to CoC. The following confounders were entered into our analysis: age; gender; indication for surgery (OA, osteonecrosis, acute femoral fracture, hip dysplasia); cup size; and head size. For all added covariates, proportional hazards assumption was visually assessed by use of log-minus-log curves.^23^ For secondary outcomes the reasons for early cup revision and the type of revision procedures performed if early cup revision was done, were expressed in numbers with accompanying proportions. This was compared between the groups using chi square tests. Separately, aseptic loosening of the cup as reason for early revision was considered endpoint in the secondary analysis. As described for the primary analysis, 2-year revision rates were calculated and compared by use of a Log Rank test and Cox proportional Hazard model. A *p*-value <0.05 was considered significant. Yearly numbers of the CoC and CoPE bearings were described to assess changes over time. Statistical analyses were performed with Statistical Package for Social Sciences (SPSS) version 26.0 (IBM Corp., Armonk, New York, USA).

### Ethical standards

The dataset and analysis were performed in compliance with the standards of the LROI regulation on research and registry data. The design and reporting of this study were done in accordance with the Strengthening the Reporting of Observational studies in Epidemiology (STROBE) statement. This research was in compliance with the Helsinki Declaration.

### Methodological safeguards to prevent bias

Only the data of those patients meeting our inclusion criteria were provided to our research team by the LROI. We analysed the data blinded. Cups were categorised in cup A, B and C, based on the three most used implant types available in the LROI with both CoC and CoPE bearing. Unblinding for manufacturer of the cups was performed after the writing of the results section.

## Results

From 2007 to 2019 a total of 326,606 THAs were registered in the LROI. In 97,013 THAs a press-fit cup was implanted with either a CoC (*N* = 17,197) or CoPE (*N* = 79,816) bearing and reached a 2-year follow-up (2007–2017). A total of 33,454 of these THAs used one of the three most used cup types available with both CoC and CoPE bearing. This group included 12,535 CoC and 20,919 CoPE THAs. The baseline characteristics of these procedures are shown in Table 1.

**Table 1.** Baseline characteristics of patients with press-fit THA performed from 2007 to 2017 in The Netherlands with 1 of the 3 most used cup types available with both CoC and CoPE bearing (*n* = 33,454).

	CoC	CoPE
	(*n* = 12,535)	(*n* = 20,919)
Gender, *n* (%)		
Male	4914 (39)	7463 (36)
Female	7596 (61)	13424 (64)
Age, mean (SD)	65.5 (9.9)	67.4 (9.8)
BMI, mean (SD) kg/m^2^	27.3 (4.4)	27.3 (4.6)
ASA, *n* (%)		
I	3748 (31.0)	4031 (19.3)
II	7153 (59.2)	14584 (69.9)
III–IV	1183 (9.8)	2252 (10.8)
Prior operation, *n* (%)	269 (2.3)	448 (2.3)
Charnley, *n* (%)*		
A	2306 (50.8)	6297 (44.0)
B	2177 (48.0)	7735 (54.0)
C	57 (1.2)	288 (2.0)
Diagnosis, *n* (%)		
Osteoarthritis	11538 (92.0)	19249 (92.0)
Other	997 (8.0)	1670 (8.0)
Smoker, *n* (%)*	741 (14.4)	1641 (11.5)
Approach, *n* (%)		
Anterior	4878 (39.5)	7333 (35.1)
Anterolateral	579 (4.7)	1123 (5.4)
Direct lateral	1694 (13.7)	2890 (13.8)
Posterolateral	5176 (42.0)	9500 (45.5)
Other	11 (0.1)	28 (0.2)
Cup type, *n* (%)		
Pinnacle, DePuySynthes	8783 (70.1)	10765 (51.5)
Exceed ABT, Zimmer-Biomet	3696 (29.5)	6769 (32.4)
Trident Tritanium, Stryker	56 (0.4)	3385 (16.2)
Cup size mm, mean (SD)	53.9 (3.4)	53.5 (3.3)
Head diameter mm, *n* (%)		
28	1050 (8.4)	4772 (22.8)
32	2038 (16.3)	12131 (58.0)
36	9447 (75.4)	4016 (19.2)

CoC, ceramic-on-ceramic; CoPE, ceramic-on-polyethylene; BMI, body mass index; SD, standard deviation.

* Numbers do not add up to total due to missing values.

### Early cup revision due to all reasons

**Figure 1.**
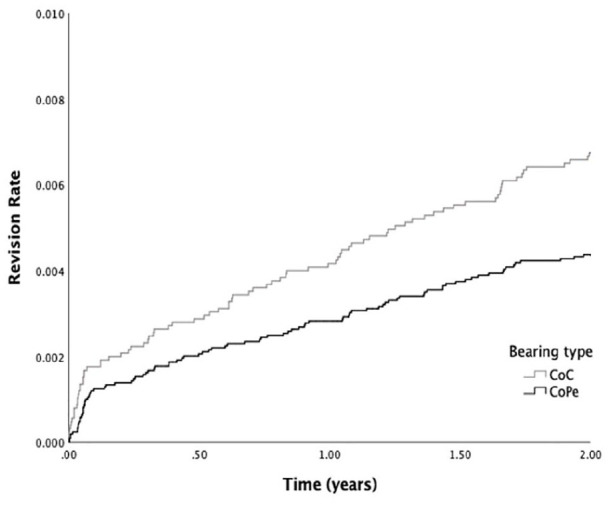
Revision rate of press-fit THA performed from 2007–2017 in The Netherlands with one of the three most used cup types between CoC (*N* *=* 12,535) and CoPE (*N* *=* 20,919) bearing.

Focused on 2-year cup revision due to all reasons, the overall 2-year cumulative cup revision rate was 0.53% [95% confidence interval (CI), 0.45–0.60]. Pooled analysis for CoC and CoPE was performed since no significant interaction between bearing and cup type was observed. A total of 84 CoC bearing THAs were revised at 2 years, resulting in a revision rate of 0.67% [95% CI, 0.54–0.81]. In CoPE 91 revisions were performed and a revision rate of 0.44% [95% CI, 0.34–0.54] was observed. The results of the Kaplan-Meier analysis are shown in Figure 1. This resulted in a significantly lower hazard of early revision in CoPE (hazard ratio [HR] 0.65 [96% CI, 0.48–0.87]) (*p* = 0.004). After adjustment for confounders (age, gender, cup type, head size) this outcome remained significant (HR 0.64 [95% CI, 0.44–0.93]) (*p* = 0.019) in favour of CoPE over CoC.

### Overall reasons for early cup revision

The reasons for early cup revision are shown in Table 2. Overall, more cup revisions due to loosening were observed in CoC than CoPE (*p* = 0.03). After adjustment for head size, Log-regression analysis showed an OR 0.398 [95% CI, 0.158–1.00] for revision due to dislocation of CoC compared to CoPE (*p* = 0.05).

**Table 2.** Reasons for revision in cup revision of press-fit THAs from 2007 to 2017 in The Netherlands, in numbers with proportions (%).

	CoC	CoPE	*p*-value
	(*n* = 84)	(*n* = 91)	
Infection	21 (25.0)	30 (33.0)	0.25
Wear of inlay	2 (2.4)	0 (0)	0.23
Periprosthetic fracture	2 (2.4)	6 (6.6)	0.28
Dislocation	22 (26.2)	25 (27.5)	0.85
Cup loosening	22 (26.2)	12 (13.2)	0.03
Periarticular ossification	0 (0)	1 (1.1)	1.00
Other	20 (23.8)	21 (23.1)	1.00
Missing	0 (0)	0 (0)	1.00

CoC, ceramic-on-ceramic; CoPE, ceramic-on-polyethylene.

Since a patient may have more than 1 reason for revision of the cup, the total can exceed 100%.

### Early cup revision due to aseptic loosening

The Kaplan-Meier analysis showed an overall 2-year cumulative cup revision rate due to aseptic loosening of 0.094% [95% CI, 0.054–0.132]. In CoC a total of 19 cup revisions due to aseptic loosening were observed, with a revision rate of 0.153% [95%CI 0.075–0.231]. CoPE showed a revision rate of 0.058% [95%CI 0.019–0.097] with a total of 12 revisions of the cup due to aseptic loosening. This difference resulted in a HR of 0.378 [95%CI 0.183–0.778] of CoPE compared to CoC (*p* = 0.007). After adjustment for confounders (head size) an HR of 0.475 [95%CI 0.197–1.141] was observed of CoPE over CoC (*p* = 0.096). The reason why there is a small difference in the numbers of cup loosening mentioned in Table 2 and the number of cup revisions due to aseptic loosening is due to the fact that loosening may also occur in cases with other reasons for revision as well, like septic revision cases.

### Incidence of revision procedures

The incidence of different cup revision procedures is shown in Table 3. Overall, the revision procedures performed did not significantly differ between CoC and CoPE (*p* = 0.09).

**Table 3.** Revision procedures performed in case of cup revision in press-fit THAs from 2007 to 2017 in The Netherlands, in numbers with proportions (%).

	CoC	CoPE
	(*n* = 84)	(*n* = 91)
Girdlestone (infection)	11 (13.1)	21 (23.1)
Cup revision	44 (52.4)	50 (54.9)
Total revision	29 (34.5)	20 (22.0)

CoC, ceramic-on-ceramic; CoPE, ceramic-on-polyethylene.

### Incidence of CoC and CoPE bearing in THA

**Figure 2.**
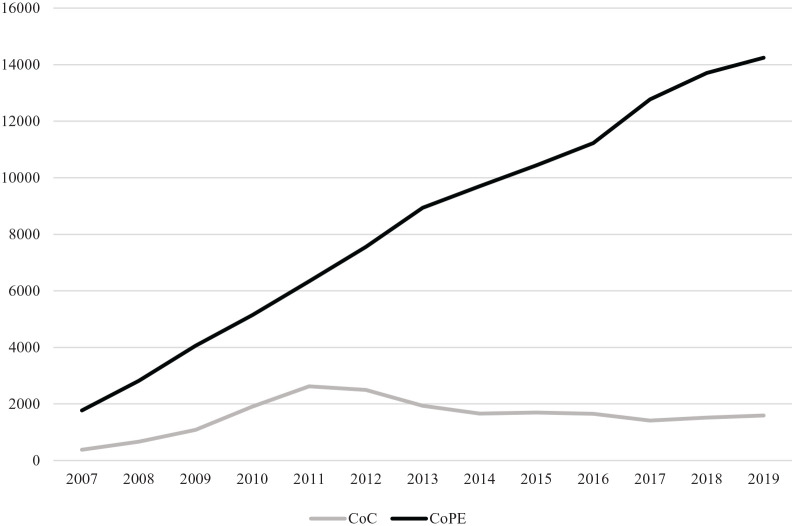
Absolute number of CoC and CoPE bearing in press-fit THA over time from 2007–2019 in The Netherlands (*N* = 129,358), horizontal axis: years; vertical axis: number of THA procedures.

In Figure 2 the absolute incidence of CoC and CoPE bearing in THAs as registered in the LROI are shown over time. From the start of the LROI in 2007 till 2011, an increase in the number of THAs performed with CoC bearing was observed. This incidence has decreased in recent years, whereas the incidence of CoPE is still increasing from the beginning of the LROI until now.

## Discussion

The main finding of this LROI observational study is an approximately 2-fold higher 2-year cup revision rate for all reasons observed in CoC. This was in line with our hypothesis. Nevertheless, early revision risk for both articulations was very low. To our knowledge, this is the first arthroplasty register study showing results focused on the 2-year cup revision risk between CoC and CoPE in THA. Moreover, recent systematic reviews have not shown significant differences in revision rates on short to mid-term either.^24,25^ The fact that both systematic reviews showed no significant difference in revision rate between bearings could be attributed to the lower number of THAs included in all separate studies and the difference in follow-up time between studies in combination with the fact that different reasons for revision occur on different time-points in both bearings.

In line with our hypothesis, the reasons for revision differed significantly between bearings. The first main reason for early revision was loosening. Our outcomes showed significantly more loosening and aseptic loosening in CoC, which was in line with our hypothesis. A recent national registry funded this with an HR of 0.65 [95% CI, 0.58–0.73] for CoC and 0.46 [95% CI, 0.38–0.55] for CoXLPE for revision due to aseptic loosening when compared to metal-on-polyethylene (MoPE) at a mean follow-up of 4.4 years.^26^ Our hypothesis is based on the fact that after uncemented cup implantation, the primary stability obtained by press-fit decreases over time. The transition to secondary stability is obtained when osseointegration becomes sufficient.^13^ Harder bearing couplings, like CoC, raise the total stiffness of the implant.^27^ In this way, the forces on the implant are less absorbed by the bearing and are transferred to the interface between the bone and the cup. We theorise that this jeopardises osseointegration and results in migration of the cup and as a result can cause failure of ingrowth of the cup and thereby aseptic loosening and revision. Focused on migration, Zhou et al.^17^ found no increased early migration in CoC compared to metal-on-cross-linked PE bearing. More randomised RSA between CoPE and CoC should be done to confirm whether migration rates are even higher in CoC, without always resulting in aseptic loosening.

The second major reason for early revision was dislocation, which showed no difference between bearings. After correction for head size, the odds for revision due to dislocation were higher in CoC, but not significantly. In CoC larger femoral head sizes are used more often, since in CoPE their use is associated with higher volumetric wear.^28^ However, the use of a bigger head size is presumed to increase range of motion, causing less impingement and as a result fewer dislocations.^29^ Another registry study observed dislocation as reason for revision at 9 years in 20% in CoC, compared to 33% in CoPE and 30% in CoHXLPE, which was declared by the use of a bigger head size in CoC.^12^ This higher risk of dislocation at long-term can be explained by its correlation with wear, which only occurs on long-term in CoPE.^30^ These results suggest that our odds of revision after correction for head size were not significant in the short-term but raise the idea that this might become significant in the longer term due to wear in CoPE.

The last main reason for early revision was infection, which did not differ between bearings. A recent systematic review reported no significant difference in rate of prosthesis infection based on the existing clinical data between bearings.^31^ Additionally, Pitto and Sedel^11^ showed no difference in revision rate due to infection within six months. Our results support this by showing no potential advantage of bearing on infection in the short term. However, the difference in Girdlestone procedures was higher in CoPE, which might be influenced by the number of cases of infection in this group. Since this procedure has an important impact on patients and the performance of THA after reimplantation, this outcome should be considered in clinical planning.

Since early cup revision is multi-factorial (e.g. patient characteristics, implant design, position, alignment, biocompatibility, microscopic structure, macroscopic design, surgical approach) it is hard to investigate a specific factor. Several confounders were seen in our study, like age, gender and cup type. Many studies have suggested that these factors can have an influence on a higher risk of overall revision, like a specific cup type, a lower age at the moment of surgery and female gender.^32–35^ Although the higher incidence of revision in CoC was still significant after correction for these confounders in our study, it shows that the aetiology of early revision is multi-factorial. Focusing on aseptic loosening, in older patients, due to the reduced quality and density of the subchondral trabecular bone, in which the cup is inserted after reaming, there may be an increase in its elasticity.^36,37^ Since a lower bone density contributes to cup migration, this can complicate achievement of sufficient primary stability for transition to secondary stability.^38^ However, osteoarthritis (OA) might change this relationship of age and quality of subchondral bone, since in late-stage OA the density, volume and thickness of the subchondral bone increases, which increases the stiffness of the bone bed for implantation.^36,39^ Moreover, bone quality is influenced by many factors, like bone mineralisation disorders, bone remodelling disorders, collagen disorders, inflammatory conditions like rheumatoid arthritis, physical activity, genetics, smoking, obesity and nutrition deficiencies.^40,41^ All the above mentioned factors could lead to impaired bone quality, which might theoretically increase the risk of aseptic loosening in combination with a stiff CoC bearing resulting in impaired osseointegration. Thus the idea is raised that it might be preferable for CoC to be used only in younger patients and patients with no impaired bone quality. Further research needs to determine if the aforementioned factors like age and OA stadium might relate to increased chance of aseptic loosening. However, most variables usually happen concurrently, which might complicate isolated research on one of these factors.

Our study showed that the incidence of THAs using CoPE is still growing and the usage of CoC is shrinking. An explanation might be that ceramic inserts are up to three times more expensive than PE.^42^ Nevertheless, CoC is more often placed in younger patients and therefore needs longer durability. Long-term cost analysis, which has not been performed between CoC and CoPE to our knowledge, needs to clarify whether differences in outcomes, complications and revision rates are cost-effective to the cost of both bearings.

### Limitations

First, since this national registry study is based on observational data, this study cannot conclude causality. Secondly, there is indication bias, which cannot be discounted when comparing different articulation combinations. Thirdly, revision due to aseptic loosening is a rare event, even in our study register; therefore, no survival analysis with correction for confounders was possible in this multi-factorial problem. Fourthly, we combined all different types of PE inserts in one group. This could influence other reasons for revision than aseptic loosening, like wear. Fifthly, wear as reason for revision was observed twice in CoC. Liner fractures are not separately reported in the LROI, which is an important shortcoming of the LROI, since this is one of the main concerns of the use of CoC. However, since wear does not occur in CoC, these two cases are most likely to have been revised due to a ceramic liner fracture. Sixthly, the use of additional screws is not separately reported in the LROI and therefore its potential confounding effect on revision has not been analysed in our study. However, studies in the literature report that screws have no effect on migration, wear and (early) revision.^43–45^ Finally, revision rates may differ from the literature since this research was focused on reasons for cup revision only and a notable group was reported as ‘other’ mentioning the reason for revision, which was not reported in the LROI.

### Implications for further research

Since the aetiology of early revision is multi-factorial, more randomised controlled studies using the same implant need to be performed to eliminate baseline variability. Moreover, more randomised controlled RSA studies need to be performed between CoC and CoPE to identify risk factors for migration and potential resulting aseptic loosening.

## Conclusion

A higher 2-year cup revision rate in press-fit THA was observed in CoC compared to CoPE. Cup loosening was the only significantly different reason for revision and seen more often in CoC and mostly aseptic. Future randomised controlled trials need to confirm causality, since the early cup revision data provided have the potential to be useful when choosing the bearing in press-fit THA, when combined with other factors like bone quality and patient and implant characteristics.
